# Efficacy and safety of direct oral anticoagulants with and without Aspirin: A systematic review and Meta-analysis

**DOI:** 10.1016/j.ijcha.2022.101016

**Published:** 2022-03-26

**Authors:** Talal Almas, Adeena Musheer, Arooba Ejaz, Fahd Niaz Shaikh, Anousheh Awais Paracha, Fizza Raza, Maryam Sarwar Khan, Fahad Masood, Faiza Siddiqui, Saamia Raza, Muhammad Fahad Wasim, Muhammad Hasnain Mankani, Kaneez Fatima, Abdul Mannan Khan Minhas

**Affiliations:** a Department of Medicine, Dow University of Health Sciences, Karachi, Pakistan; b Department of Medicine, Baqai Medical University, Karachi, Pakistan; c Department of Medicine, Aga Khan University Hospital, Karachi, Pakistan; d Department of Medicine, RCSI University of Medicine and Health Sciences, Dublin, Ireland; e Department of Internal Medicine, Forrest General Hospital, Hattiesburg, MS, United States

**Keywords:** Anticoagulants, Bleeding, Aspirin, Stroke, Hospitalization

## Abstract

**Background:**

Various anticoagulant therapies are prescribed to patients under physicians’ discretion and recently Direct Oral Anticoagulants(DOAC) have been under trials to evaluate their safety and efficacy. In addition to this, the regimen of DOACs and Aspirin is of keen interest as researchers continue to find an optimal regimen to treat blood clots in patients. This study is a systematic review and *meta*-analysis of randomized controlled trials and observational studies that asses the safety and efficacy of DOAC with and without Aspirin.

**Methods:**

We queried MEDLINE and Cochrane CENTRAL from their inception to April 2021, for published and randomized controlled trials and observational studies in any language that compared dual (DOAC + ASA) therapy or mono (DOAC alone) therapy in patients with AF. The results from the studies were presented as risk ratios (RRs) with 95% confidence intervals (CIs) and were pooled using a random-effects model. Endpoints of interest included major bleeding, myocardial infarction (MI), major adverse cardiovascular events (MACEs), hospitalizations, all-cause mortality, and stroke.

**Results:**

The risk of major bleeding was significantly lower in the DOAC alone group compared with DOAC plus aspirin group. Non-significant results were obtained (P value greater than 0.05) for other outcomes establishing that DOAC monotherapy was not superior to the combined regimen in reducing the risk of MACE, Stroke, Hospitalization, Death.

**Conclusion:**

Among patients with NVAF (Non valvular Atrial Fibrillation) and VTE (Venous thromboembolism) receiving anticoagulation prophylaxis, in terms of safety profile our comparisons showed a statistically significant reduction in Major Bleeding in DOAC Alone group compared with DOAC Plus Aspirin.

## 1 Introduction

Long-term anticoagulant therapy is the standard of care to prevent the complications of atrial fibrillation (AF). Currently, four direct oral anticoagulants (DOACs) (dabigatran, rivaroxaban, apixaban, and edoxaban) are recommended to achieve better safety and efficacy outcomes.[1] In 2019, DOACs accounted for 74% of all anticoagulants prescribed for National Health Services (NHS) practices in England.[2] In the past, warfarin, a vitamin K antagonist (VKA) together with antiplatelet therapy (APT) was common for therapeutic purposes in patients with atrial fibrillation, venous thromboembolism, and atherosclerosis. However, VKA plus APT increases the risk of major bleeding by two- to four-fold compared with VKA alone.[3] Recent evidence has favored Direct oral anticoagulants (DOACs) over VKAs due to better safety outcomes.[4] The risk of major bleeding makes the management of patients challenging showing that it is imperative to find an anticoagulant regimen that provides ideal efficacy and safety outcomes.

More recently, randomized control trials and cohort studies compared an alternative therapy- monotherapy with DOAC alone vs dual therapy with DOAC + Aspirin (ASA) to identify the safest treatment option for patients with atrial fibrillation (AF).[3], [5], [6] Evidence shows that patients exposed to dual therapy have a higher risk of bleeding and major adverse cardiac events.[3] Nevertheless, there are limited *meta*-analyses and systematic reviews that investigate the safety and efficacy of dual therapy (DOAC + ASA) vs mono therapy (DOAC alone) among patients with NVAF and VTE (Venous thromboembolism).

Majority of the earlier studies had limited follow-up duration and included various types of DOACs due to which the effect of therapy on variable outcomes was not apparent. Therefore, pooling results from several studies can help provide a better assessment of the outcomes. Hence, we conducted a *meta*-analysis of randomized controlled trials and cohort studies to address the question of which therapy- dual therapy (DOAC + ASA) or mono therapy (DOAC alone)- is most suitable for the management of patients with AF.

## 2 Materials and Methods

### 2.1 Data sources and strategy

This *meta*-analysis was performed in accordance with the Preferred Reporting Items for Systematic review and Meta-Analyses (PRISMA) guidelines.[7]

### 2.2 Study selection

Two independent reviewers (FNS and AE) performed an electronic search of MEDLINE and Cochrane CENTRAL from their inception to April 2021 using an extensive search strategy which involved all possible generic, pharmaceutical and trade names and abbreviations of the drugs along with MeSH terms and Boolean operators ‘AND’ and ‘OR’. The search strategy is included in Supplemental Table 1.

**Table1:** Characteristics of Included Trials.

RCTs		
Characteristics	**COMPASS**	**AFIRE**
Trial Name	Rivaroxaban with or without Aspirin in Stable Cardiovascular Disease	Antithrombotic Therapy for AF with Stable Coronary Disease
Patients, n	27,395	2236
Enrollment initiation	2013	2015
Enrollment completion	2016	2017
Year of publication	2017	2019
Population	Patients with stable atherosclerotic vascular disease	Patients with atrial fibrillation who had undergone PCI or CABG more than 1 year earlier or who had angiographically confirmed coronary artery disease not requiring revascularization to receive monotherapy with rivaroxaban or combination therapy with rivaroxaban plus a single antiplatelet agent
Trial Type	Double-blind, double-dummy	Multicenter, open-label
Inclusion criteria	Patients with CAD, PAD, or both. CAD patients < 65 y of age were also required to have documentation of atherosclerosis involving at least two vascular beds or to have at least two additional risk factors	Age ≥ 20 y had received a diagnosis of AF and stable CAD The patients were required to have a score of at least 1 on the CHADS_2_ scale at least one of the following criteria a history of PCI, including angioplasty with or without stenting, at least 1 year before enrolment a history of angiographically confirmed CAD (with stenosis of ≥ 50%) not requiring revascularization or a history CABG at least 1 year before enrollment
Exclusion criteria	High bleeding risk a recent stroke or previous hemorrhagic or lacunar stroke severe heart failure advanced stable kidney disease (estimated GFR < 15 ml/minute) the use of DAPT, anticoagulation, or other antithrombotic therapy noncardiovascular conditions deemed by the investigator to be associated with a poor prognosis patients receiving a proton-pump inhibitor were not eligible for the pantoprazole randomization	A history of stent thrombosis coexisting active tumor poorly controlled hypertension
Treatments	Rivaroxaban (2.5 mg twice daily) plus Aspirin (100 mg once daily) Rivaroxaban (5 mg twice daily) with an aspirin-matched placebo once daily or aspirin (100 mg once daily) with a rivaroxaban matched placebo twice daily.	Monotherapy with rivaroxaban (10 mg once daily for patients with a creatinine clearance of 15 to 49 ml per minute or 15 mg once daily for patients with a creatinine clearance of ≥ 50 ml per minute) or combination therapy with rivaroxaban at the previously stated doses plus an antiplatelet agent (either aspirin or a P2Y12 inhibitor, according to the discretion of the treating physician).
Primary efficacy outcome	Composite of cardiovascular death, stroke, or MI	Composite of stroke, systemic embolism, MI, unstable angina requiring revascularization, or death from any cause
Follow up	Mean 23 months	Median 24.1 months

Table1. Characteristics of Included Trials (continued)						
Observational Studies						

Characteristic	**Schaefer**	**Said**	**Tinkham**	**Davidson**	**Steinberg**	**Ruiz**
Trial Name	Impact of Adding Aspirin to direct oral anticoagulant w/o an apparent Indication	Concomitant use of direct oral anticoagulants and aspirin versus direct oral anticoagulants alone in atrial fibrillation and flutter a retrospective cohort	Direct oral anticoagulant plus antiplatelet therapy prescribing practices and bleeding outcomes	Bleeding Risk of Patients With Acute Venous Thromboembolism Taking Nonsteroidal Anti-Inflammatory Drugs or Aspirin	Use and Associated Risks of Concomitant Aspirin Therapy With Oral Anticoagulation in Patients With Atrial Fibrillation	Effect of concomitant antiplatelet therapy in patients with nonvalvular atrial fibrillation initiating non‐vitamin K antagonists
Patients, n	2045	6004	407	8246	7347	2361
Initiation	2009	2010	2017	2007	2010	2013
Completion	2019	2015	2017	2009	2011	2016
Year of publication	2019	2020	2019	2014	2013	2019
Population	Adults on DOAC therapy for NVAF or VTE	Patients with AF or AFL	Patients receiving DOAC therapy were evaluated for APT use at the time of hospital discharge	Patients with VTE	AF patients on OAC	NVAF patients
Inclusion crieria	Adults on DOAC therapy(apixaban, dabigatran, edoxaban and rivaroxaban) for NVAF or VTE	18 ≤ Age ≤ 100 documented AF or AFL patients taking one of the following DOACs apixaban, rivaroxaban, or dabigatran	Age ≥ 18 documented DOAC use	Patients with VTE receiving study anticoagulant therapy combined with either NSAID or aspirin compared to patients receiving anticoagulant therapy only	Age ≥ 18 able to provide follow up every 6 months	NVAF patients receiving a first NOAC prescription for the prevention of stroke or systemic embolism
Exclusion criteria	History of heart valve replacement, recent MI, or<3 months of follow-up	Patients with valvular AF were excluded history of VTE such as DVT or PE patients who were taking different antiplatelets such as P2Y12 inhibitors were also excluded.	DOAC for VTE prophylaxis following orthopedic surgery were excluded due to the short duration of therapy.	Patients with a clearly increased bleeding risk	Patients with reversible causes of AF (eg, thyroid disease, postoperative AF) or patients with a life expectancy of < 6months patients receiving other antiplatelet drugs	Patients with AF who received OAC for other indications history of NOAC therapy patients with an ACS, PCI with stent implantation or ischaemic stroke within the last 12 months or with a percutaneous intervention with stent implantation in a noncoronary artery in the previous month
Treatments	DOAC+ASA vs DOAC (apixaban, dabigatran, edoxaban and rivaroxaban)	DOAC+ASA vs DOAC (apixaban, rivaroxaban, or dabigatran)	DOAC+APT vs DOAC monotherapy (most common apixaban and rivaroxaban)	DOAC+Aspirin vs DOAC (Rivaroxaban)	DOAC+ASA	DOAC+ASA vs DOAC therapy
Definition of primary bleeding outcome	Any new bleeding event	MACE defined as ACS, ischemic strokes, and embolic events	Bleeding was categorized as major or CRNMB using definitions of the ISTH	Major bleeding fatal occurred at a critical site associated with a decrease in hemoglobin concentration greater than 2 g/dL and/or the need for transfusion of at least 2 units of RBCs. Clinically relevant nonmajor bleeding not major but associated withmedical intervention unscheduled contact with a physician (temporary) cessation of study treatment discomfort for the patient such as pain or impairment of activities of daily living	6-month bleeding, hospitalization, ischemic events, and mortality	Major bleeding which was defined according to 2005 ISTH criteria
Follow up	15.2 months	Minimum 2 years	6 months	3, 6, 12 months	6 months	3 months

AF, Atrial fibrillation, PCI, Percutaneous Coronary Intervention, CABG, Coronary artery bypass graft, CAD, Coronary artery disease, PAD, Peripheral artery disease, GFR, Glomerular filtration rate, DAPT, Dual antiplatelet therapy, MI, Myocardial infarction, ISTH, International Society on Thrombosis and Haemostasis (ISTH), DOAC, Direct oral anticoagulant, VTE, Venous thromboembolic disease, DVT, Deep vein thrombosis, PE, Pulmonary embolism, AFL, Atrial flutter, MACE, Major adverse cardiac events, ACS, Acute coronary syndromes, CRNMB, Clinically relevant non-major bleeding

The predefined eligibility criteria for our *meta*-analysis were: (a) published and randomized controlled trials (RCTs) or Observational Studies; (b) adult patients (≥18 years) with follow up of minimum 3 months (c) compared DOAC alone vs DOAC plus aspirin with respect to safety and efficacy outcomes (d) reported at least one of the following outcomes of: major bleeding, MI, MACE, hospitalizations, all-cause mortality, stroke or composite of any of these listed outcomes. Major bleeding and MACE definitions varied across individual studies but were accepted due to the scant pool of studies available. Any dispute between the two independent reviewers (FNS and AE) regarding study selection was resolved by discussion and a mutual consensus with a senior investigator (AM).

### 2.3 Data extraction and quality assessment of studies

**Fig. 1:**
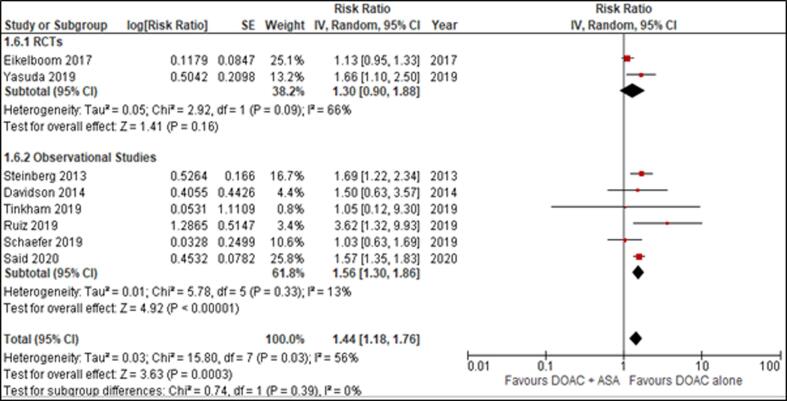
Effect of DOAC + ASA versus DOAC alone on Major Bleeding.

The studies yielded by our search strategy were cross verified by the two independent reviewers (FNS and AE) and compiled in Endnote Reference Library (Version X7.5; Clarivate Analytics, Philadelphia, Pennsylvania) software where duplicates were searched and removed. All the full texts of the remaining articles were then thoroughly reviewed to extract the following outcomes and their RRs. In addition, the reference sections of these full-text articles were also manually screened for any relevant studies that might have been missed out during the electronic search. In cases where raw data was available, the summary events were proportionated to calculate RRs with 95% confidence intervals (CIs). Effect sizes such as HRs (Hazard Ratios) were also treated as RRs (Risk Ratios). Moreover, study characteristics and patient baseline characteristics were also extracted and reported in Table 1 of the text and Supplementary Table 3 respectively. To assess the quality of studies across six domains (selection bias, performance bias, detection bias, attrition bias, reporting bias, and other bias), we used Newcastle-Ottowa scale for observational studies and Cochrane Collaboration’s risk of bias tool for RCTs[8], [9] results of which are reported in Supplemental Table 2 and, Supplemental Figure 1a and 1b, respectively.Fig. 1

### 2.4 Statistical analysis

All statistical analyses were carried out using RevMan (version 5.3; Copenhagen: The Nordic Cochrane Centre, The Cochrane Collaboration). The forest plots of relevant outcomes were visually represented after the RRs with 95% CIs were pooled using the random- effects model. These outcomes were then stratified into two subgroups based on study type (RCT or observational) and the chi-square test was performed to evaluate the differences between the subgroups. In addition, heterogeneity in effect sizes was assessed using Higgin’s I[2] statistics where I[2] value of greater than 50 % was considered significant.[10] Lastly, we also performed the Begg’s test and graphed funnel plots to check for any publication irregularities. P-value < 0.05 was considered significant for all the above analyses.[11]

## 3 Results

**Fig. 2:**
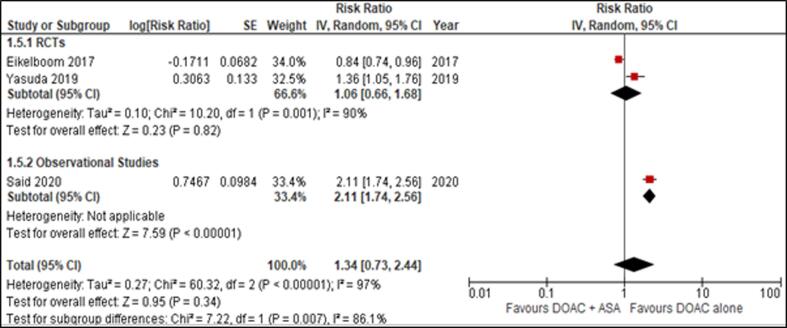
Effect of DOAC + ASA versus DOAC alone on MACE.

We selected 9 studies (2 RCTS and 7 Observational studies) out of the 2781 articles we reviewed for eligibility (Supplemental Figure 2). COMPASS (RCT) assigned 27,395 patients with rivaroxaban plus aspirin, rivaroxaban, or aspirin while AFIRE assigned subjects with Rivaroxaban or Combination Therapy with an antiplatelet agent. PIONEER AF PCI (observational study) assigned participants low-dose rivaroxaban plus a P2Y12 inhibitor, rivaroxaban plus DAPT, or vitamin K antagonist plus DAPT. Schaefer (registry-based cohort study) assigned patients with DOAC plus ASA and DOAC only. Tinkham assigned patients to DOAC monotherapy or DOAC + APT. Said (Observational) randomly assigned patients to DOAC monotherapy and DOAC with concurrent aspirin. Davidson (observational study) randomly assigned 8246 patients with combined anticoagulant therapy with and without aspirin. Steinberg (observational study) assigned subjects with OACs only or OAC + Aspirin. Ruiz (retrospective multicenter study) assigned patients to concomitant APT (DOAC + ASA) or DOAC alone.Fig. 2

### 3.1 Outcomes

#### 3.1.1 Major bleeding

Data for the primary safety outcome of major bleeding was provided in all the included studies. The risk of major bleeding was significantly lower in the DOAC alone group compared to the DOAC plus aspirin group (RR = 1.44 [1.18, 1.76]; p < .001), with moderate statistical heterogeneity (I^2^ = 56%) (Fig. 1). Fig. 6 is a graphical illustration indicating a general trend of increased bleeding risk in DOAC plus aspirin group across varying follow-up time durations in the included studies.

#### 3.1.2 Major adverse cardiovascular events

Data for MACE, reported by 3 studies, yielded non-significant results establishing that DOAC monotherapy was not superior to the combined regimen in reducing the risk of cardiovascular complications (RR = 1.34 [0.73, 2.44]) (Fig. 2). Sensitivity analysis was done by excluding the COMPASS trial and no significant interaction was noted between the two treatment groups and the risk of developing MACE, which is consistent with the findings of the primary analysis.

#### 3.1.3 Stroke

Stroke was reported by four studies. No statistically significant relationship was illustrated between the two treatment groups and the risk of developing stroke (RR = 1.26 [0.50, 3.14]) (Fig. 5*)*. However, on sensitivity analysis, the use of DOAC agents alone was associated with a statistically significant reduction in the risk of stroke (RR = 2.16 [1.55, 3.01]; p < .001) and a low level of heterogeneity was observed (I^2^ = 5%).

#### 3.1.4 Hospitalization and death

**Fig. 3:**
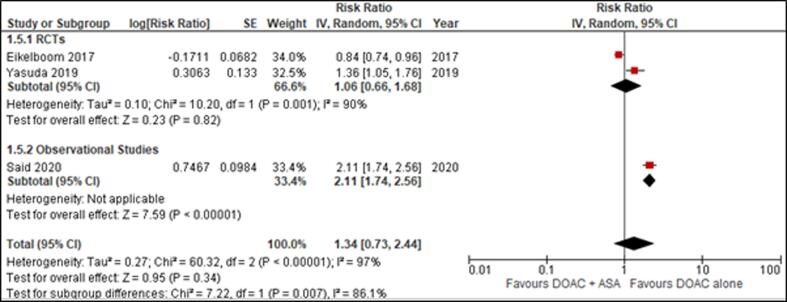
Effect of DOAC + ASA versus DOAC alone on Hospitalization.

**Fig. 4:**
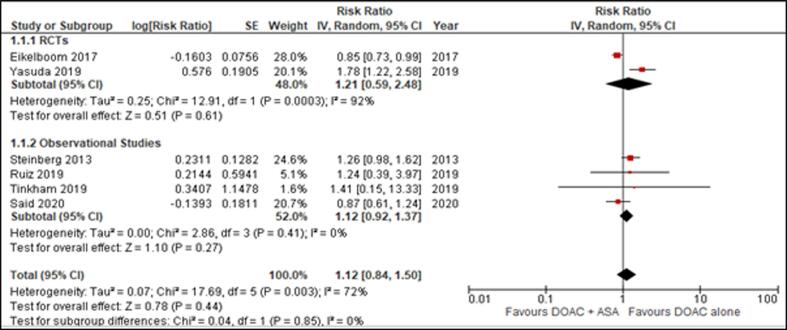
Effect of DOAC + ASA versus DOAC alone on Death.

Data for hospitalization was provided in 3 studies, whereas 6 studies reported death. The rate of hospitalization was similar across both treatment groups (RR = 1.06 [0.97, 1.14]) (Fig. 3*)*. Likewise, death occurred at a comparable rate between the experimental and the control arm, producing non-significant results (RR = 1.17 [0.86, 1.60]; p = .31) (Fig. 4*)*. Additionally, death as an outcome also demonstrated a significant trend in favor of DOAC monotherapy (RR = 1.33 [1.05, 1.68]; p = 0.02; I^2^ = 19%).

**Fig. 5:**
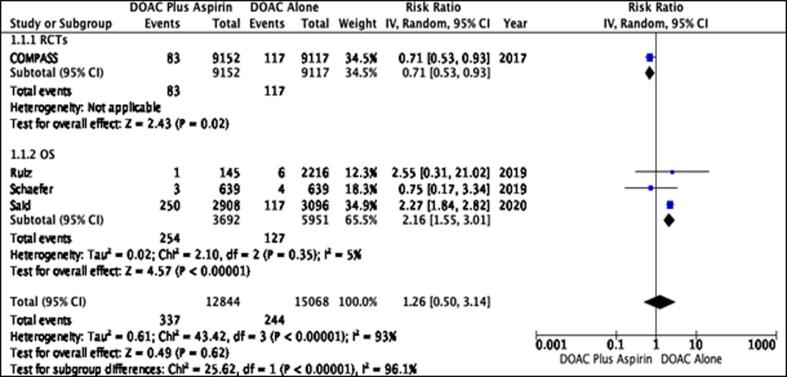
Effect of DOAC + ASA versus DOAC alone on Stroke.

**Fig. 6:**
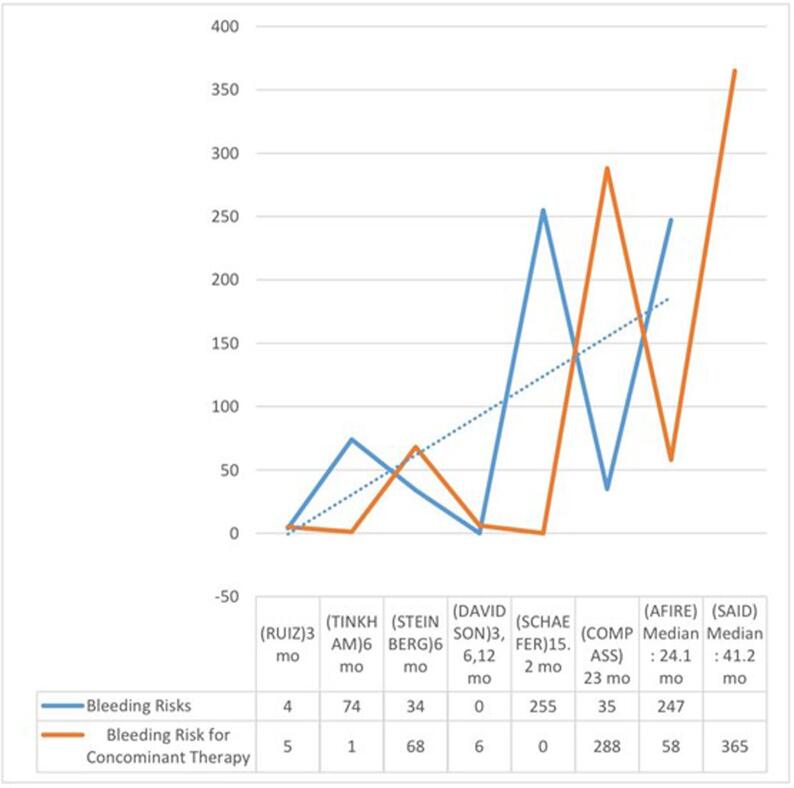
General Trend indicating the association between DOAC plus aspirin therapy and bleeding risk across different follow-up times.

## 4 Discussion

In this extensive *meta*-analysis comprising more than 50 000 patients, DOAC monotherapy significantly reduced the risk for major bleeding compared with combination therapy with DOAC and ASA. Our findings remained consistent when studies were further analyzed according to specific DOAC indications. There was no statistically significant difference in effect on risks for hospitalization, death, and ischemic endpoints like major adverse cardiovascular events (MACE), myocardial infarction (MI), and stroke between the two groups.

Our results reflect the outcome trend in the 2 included RCTs and 5 observational studies. In the COMPASS trial[5], there was frequent occurrence of major bleeding in patients in the rivaroxaban plus ASA group 3.1% versus rivaroxaban alone group 2.8%.[5] The AFIRE trial reported the superiority of rivaroxaban monotherapy to combination therapy for the primary safety end point of major bleeding, with event rates of 1.62% and 2.76% per patient year respectively.[3] In Said et al study, bleeding occurred more in the DOAC + ASA group 19.3% compared to the DOAC only group 11.8%.[6] In the study by Ruiz et al, concomitant ASA was associated with higher rates of bleeding with no benefits in terms of ischemic protection.[12] The findings of Davidson et al study aligns with the findings of the previous studies that demonstrate that concomitant use of ASA is associated with an increased risk of major bleeding.[13] Tinkham et al study showed non-significant results for major bleeding between DOAC + APT and DOAC monotherapy. This could have occurred largely since the sample size of this study was relatively small and the follow up duration was limited. Hence, the conclusion regarding the safety of DOAC + APT cannot be deduced from this study.[14] The PIONEER AF-PCI trial showed similar outcome trends; however, since the trial included an additional intervention, P2Y12 inhibitor, we could not pool results from this trial.[15] It assessed the efficacy and safety of DOAC and ASA along with P2Y12 inhibitor; thus, it did not reflect the outcome trends associated with the use of DOAC plus ASA only.

In the prior *meta*-analysis by Kumar et al[16] combination therapy of ASA/antiplatelet drug with DOAC resulted in higher rates of bleeding compared to DOAC alone regardless of the type of DOAC investigated which was consistent with the finding of our *meta*-analysis. The Lopes et al[17] network *meta*-analysis investigated the safety and efficacy of antithrombotic regimen in patients which demonstrated that DOAC plus P2Y12 inhibitor regimen results in less bleeding compared with VKA and DAPT. However, it also addressed that omitting ASA from the antiplatelet strategies resulted in less bleeding outcomes, without significant difference in MACE, compared with strategies including ASA.

The impact of concomitant antiplatelet therapy with oral anticoagulants in the incidence of bleeding and ischemic events has already been studied in patients receiving VKA.[18] The studies have been unsuccessful in demonstrating a benefit in terms of ischemic events prevention with VKA use, while showing an association with higher bleeding complications on the other side.[19] Bennaghmouch et al showed that it is both safer and more effective to use DOACs in comparison with VKA to treat patients with non-valvular AF and concomitant aspirin therapy.[20]

A previous network *meta*-analysis by Altoukhi et al showed interesting findings about the types DOACs in terms of safety and efficacy outcomes. The regimen of Dabigatran was placed first in reducing death from any cause whereas the regimen of apixaban came out to be superior in reduction of the risk of major or CRNM bleeding and stroke. For reduction in the risk of MI and stent thrombosis rivaroxaban regimen was to be preferred. In accordance with this ranking, VKA on the other hand, was categorized as the lowest as compared to all DOACs’ dual anti-platelet therapy regimens in terms of bleeding, MI and death.

While previous *meta*-analyses have investigated safety outcomes between DOAC monotherapy and dual antithrombic therapy, to the best of our knowledge, our *meta*-analysis is the first original study demonstrating significantly lower major bleeding events in AF patients receiving DOCs alone compared with those receiving combination therapy of DOACs + ASA. In addition to this, we further explored the correlation between aspirin therapy duration and bleeding risk, and found that prolonged aspirin therapy was linked with increased major bleeding risk. Said etal. reportedly had the highest follow-up period of 24.1 months, and a corresponding heightened bleeding risk with concomitant therapy group. These findings can be of critical value to clinicians while designing future regimens for AF patients.

The AFIRE and COMPASS trials demonstrated conflicting findings of cardiovascular events and all-cause mortality with DOAC monotherapy. While the AFIRE trial represented a significant decrease in the incidence of cardiovascular events and death from any cause in the monotherapy group, the COMPASS trial showed the contrary. Similarly, our *meta*-analysis demonstrated a non-significant reduction in cardiovascular events and all-cause mortality. Hence, it is crucial to understand the limitations of these individual studies. The COMPASS trial enrolled patients with a high risk for (possibly, recurrent) cardiovascular events, more than 60% of which had previously undergone MI. This inevitably increased the chances of success of administration of intensified antithrombotic therapy alongside ASA, leading to a 1.3% reduction in primary efficacy outcomes of MACE. (12) The trial was also prematurely terminated after consistent difference was observed in the primary efficacy outcome of cardiovascular death, stroke, and myocardial infarction in favor of rivaroxaban plus ASA. Moreover, the dosing regimens of rivaroxaban in both COMPASS and AFIRE were less than the globally approved 20 mg daily dose. In the AFIRE trial, the antiplatelet therapy was unspecified, with some patients being assigned to ASA while others were given a P2Y12 inhibitor.

Other limitations include the presence of confounding bias inherently present in observational analyses due to lack of randomization, limited follow-up duration, low rates of study events, and insufficient power to identify differences in cardiovascular and mortality outcomes between the two groups. Also, in our *meta*-analysis, results from studies using various types of DOACs were pooled together on the assumption that all DOACs are comparable in terms of safety and efficacy. However, this may not be true as demonstrated by Altoukhi et al[21] study which ranked the different types of DOACs as per their efficacy and safety. In Ruiz et al, out of the 145 patients who received concomitant antiplatelet therapy, 79 percent of them used ASA whereas 21 percent of them were given P2Y12 inhibitor, hence the results of this study do not solely reflect the safety and efficacy of ASA alone but rather the antiplatelet therapy in general. However, since this study enrolled a relatively fewer number of participants, the chances of this affecting the results are very low.

In the light of the current evidence, it becomes vital that in the non-acute setting the indication for ASA in patients with AF who are on DOAC therapy should be assessed carefully and the associated risk of bleeding should be evaluated while making efforts to minimize it wherever possible. Our results further emphasize on the need to carefully weigh the risks and benefits of initiating and/or continuing ASA therapy especially in the setting of concurrent DOAC use for AF, AFL or VTE.

The included RCTs in our *meta*-analysis had clinical and methodological heterogeneities. In the AFIRE trial there was a higher risk of all-cause mortality in the rivaroxoban + ASA group, therefore it was recommended by the independent data and safety monitoring committee to prematurely terminate the trial which may have led to overestimation of the efficacy data affecting the results of our meta- analysis. As mentioned above, the COMPASS trial was also terminated early. In addition to this, variations in baseline characteristics, including different percentages of men and women, age, comorbidities, baseline risk severity of enrolled patients across the included studies, limit our interpretation, although a random-effects model was used to reduce heterogeneity. Other factors that further contribute to between-study heterogeneity include type of DOAC, dose and duration of ASA, open-label design, and duration of follow up.

## 5 Conclusion

In conclusion, this *meta*-analysis of over 50 000 patients demonstrates that DOAC monotherapy significantly reduces the risk of major bleeding. The use of DOAC monotherapy versus combination therapy with DOAC and ASA showed inconclusive effects for all-cause mortality and ischemic outcomes. Furthermore, our results reinforce those patients with cardiovascular disease, regardless of DOAC indication, may not benefit from combination therapy with regards to primary safety end point.

## Declaration of Competing Interest

The authors declare that they have no known competing financial interests or personal relationships that could have appeared to influence the work reported in this paper.
