# Supplementary material for: Efficacy and safety of direct oral anticoagulants with and without Aspirin: A systematic review and Meta-analysis
Source: Int J Cardiol Heart Vasc. 2022 Mar 26;40:101016. doi: 10.1016/j.ijcha.2022.101016 (PMC8958538; doi:10.1016/j.ijcha.2022.101016)
Supplement: Supplementary data 1 [file mmc1.docx]

**SUPPLEMENTARY MATERIAL**

**Supplemental Table 1**. Search strategy used in each database searched

| **Database (Articles Retrieved)** | **Search Strategy** |
| --- | --- |
|  | ((("direct"[All Fields] OR "directed"[All Fields] OR "directing"[All Fields] OR "direction"[All Fields] OR "directional"[All Fields] OR "directions"[All Fields] OR "directivities"[All Fields] OR "directivity"[All Fields] OR "directs"[All Fields]) AND ("mouth"[MeSH Terms] OR "mouth"[All Fields] OR "oral"[All Fields]) AND ("anticoagulants"[Pharmacological Action] OR "anticoagulants"[MeSH Terms] OR "anticoagulants"[All Fields] OR "anticoagulant"[All Fields] OR "anticoagulate"[All Fields] OR "anticoagulated"[All Fields] OR "anticoagulating"[All Fields] OR "anticoagulation"[All Fields] OR "anticoagulations"[All Fields] OR "anticoagulative"[All Fields])) OR "DOACs"[All Fields] OR (("novel"[All Fields] OR "novel s"[All Fields] OR "novels"[All Fields]) AND ("mouth"[MeSH Terms] OR "mouth"[All Fields] OR "oral"[All Fields]) AND ("anticoagulants"[Pharmacological Action] OR "anticoagulants"[MeSH Terms] OR "anticoagulants"[All Fields] OR "anticoagulant"[All Fields] OR "anticoagulate"[All Fields] OR "anticoagulated"[All Fields] OR "anticoagulating"[All Fields] OR "anticoagulation"[All Fields] OR "anticoagulations"[All Fields] OR "anticoagulative"[All Fields])) OR ("New"[All Fields] AND ("mouth"[MeSH Terms] OR "mouth"[All Fields] OR "oral"[All Fields]) AND ("anticoagulants"[Pharmacological Action] OR "anticoagulants"[MeSH Terms] OR "anticoagulants"[All Fields] OR "anticoagulant"[All Fields] OR "anticoagulate"[All Fields] OR "anticoagulated"[All Fields] OR "anticoagulating"[All Fields] OR "anticoagulation"[All Fields] OR "anticoagulations"[All Fields] OR "anticoagulative"[All Fields])) OR ("Non"[All Fields] AND ("vitamin k"[MeSH Terms] OR "vitamin k"[All Fields]) AND ("antagonist"[All Fields] OR "antagonists and inhibitors"[MeSH Subheading] OR ("antagonists"[All Fields] AND "inhibitors"[All Fields]) OR "antagonists and inhibitors"[All Fields] OR "antagonists"[All Fields]) AND ("mouth"[MeSH Terms] OR "mouth"[All Fields] OR "oral"[All Fields]) AND ("anticoagulants"[Pharmacological Action] OR "anticoagulants"[MeSH Terms] OR "anticoagulants"[All Fields] OR "anticoagulant"[All Fields] OR "anticoagulate"[All Fields] OR "anticoagulated"[All Fields] OR "anticoagulating"[All Fields] OR "anticoagulation"[All Fields] OR "anticoagulations"[All Fields] OR "anticoagulative"[All Fields])) OR ("n 4 oleylcytosine arabinoside"[Supplementary Concept] OR "n 4 oleylcytosine arabinoside"[All Fields] OR "noac"[All Fields]) OR ("Target-specific"[All Fields] AND ("mouth"[MeSH Terms] OR "mouth"[All Fields] OR "oral"[All Fields]) AND ("anticoagulants"[Pharmacological Action] OR "anticoagulants"[MeSH Terms] OR "anticoagulants"[All Fields] OR "anticoagulant"[All Fields] OR "anticoagulate"[All Fields] OR "anticoagulated"[All Fields] OR "anticoagulating"[All Fields] OR "anticoagulation"[All Fields] OR "anticoagulations"[All Fields] OR "anticoagulative"[All Fields])) OR ("tsoac"[All Fields] OR "tsoacs"[All Fields]) OR ("factor xa inhibitors"[Pharmacological Action] OR "factor xa inhibitors"[MeSH Terms] OR ("factor"[All Fields] AND "xa"[All Fields] AND "inhibitors"[All Fields]) OR "factor xa inhibitors"[All Fields]) OR ("rivaroxaban"[MeSH Terms] OR "rivaroxaban"[All Fields]) OR ("apixaban"[Supplementary Concept] OR "apixaban"[All Fields] OR "apixaban s"[All Fields]) OR ("edoxaban"[Supplementary Concept] OR "edoxaban"[All Fields]) OR ("dabigatran"[MeSH Terms] OR "dabigatran"[All Fields] OR "dabigatran s"[All Fields]) OR ("betrixaban"[Supplementary Concept] OR "betrixaban"[All Fields])) AND ("aspirin"[MeSH Terms] OR "aspirin"[All Fields] OR "aspirins"[All Fields] OR "aspirin s"[All Fields] OR "aspirine"[All Fields] OR (("acetyl"[All Fields] OR "acetylate"[All Fields] OR "acetylated"[All Fields] OR "acetylates"[All Fields] OR "acetylating"[All Fields] OR "acetylation"[MeSH Terms] OR "acetylation"[All Fields] OR "acetylations"[All Fields] OR "acetyls"[All Fields]) AND ("salicylic acid"[MeSH Terms] OR ("salicylic"[All Fields] AND "acid"[All Fields]) OR "salicylic acid"[All Fields])) OR ("o-acetyl"[All Fields] AND ("salicylic acid"[MeSH Terms] OR ("salicylic"[All Fields] AND "acid"[All Fields]) OR "salicylic acid"[All Fields])) OR ("aspirin"[MeSH Terms] OR "aspirin"[All Fields] OR "2 acetoxybenzoic acid"[All Fields]) OR "ASA"[All Fields]) |
| Cochrane Library  (776 results) | (Direct oral anticoagulants OR DOACs OR Novel oral anticoagulants OR New oral anticoagulants OR Non vitamin K antagonist oral anticoagulants OR noac OR Target-specific oral anticoagulants OR TSOACs OR Factor Xa inhibitors OR rivaroxaban OR apixaban OR edoxaban OR dabigatran OR betrixaban) AND (Aspirin OR acetyl salicylic acid OR o-acetyl salicylic acid OR 2-acetoxybenzoic acid OR ASA) |


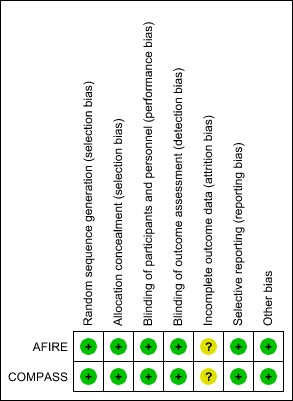

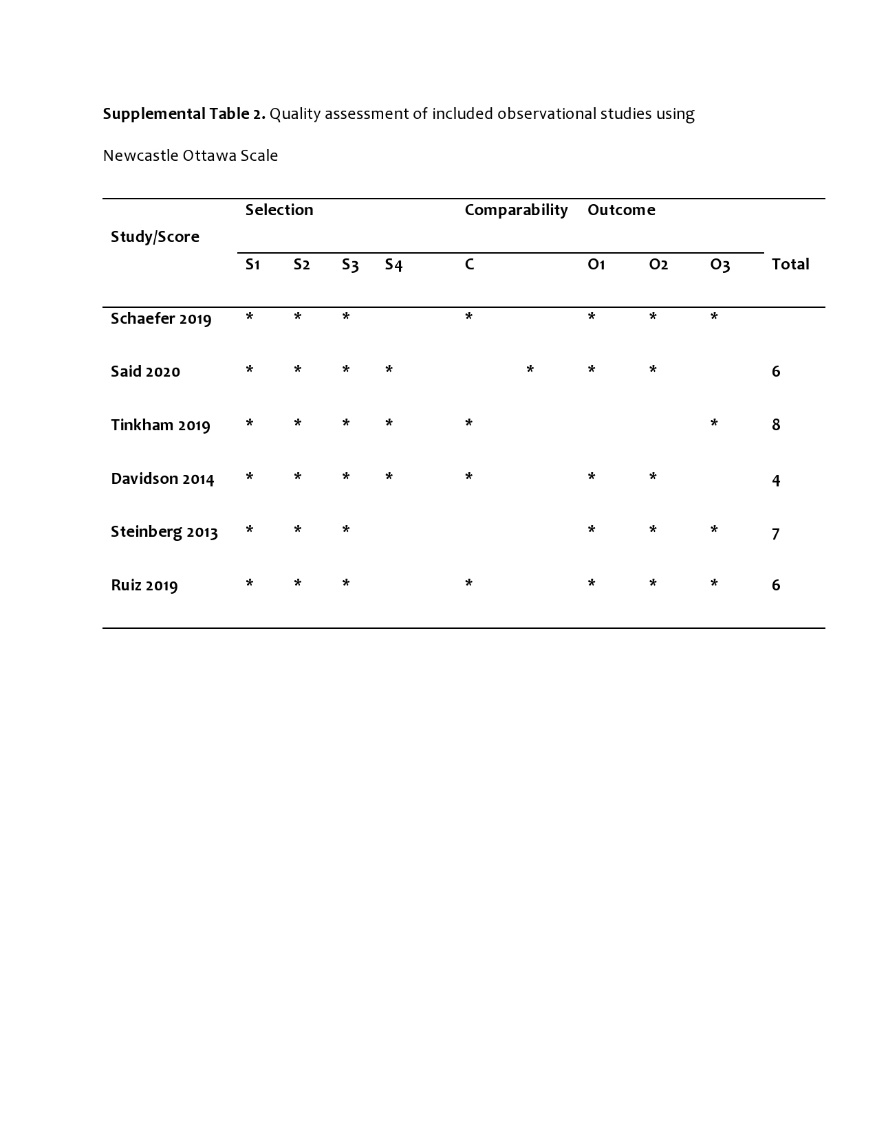


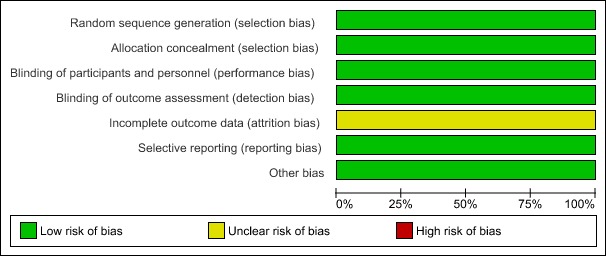


**Supplemental** Figure 1a. Quality Assessment of included Randomized Controlled Trials using Cochrane Risk of Bias Tool

**Supplemental Figure 1b**. Quality Assessment of included Randomized Controlled Trials using Cochrane Risk of Bias Tool

| 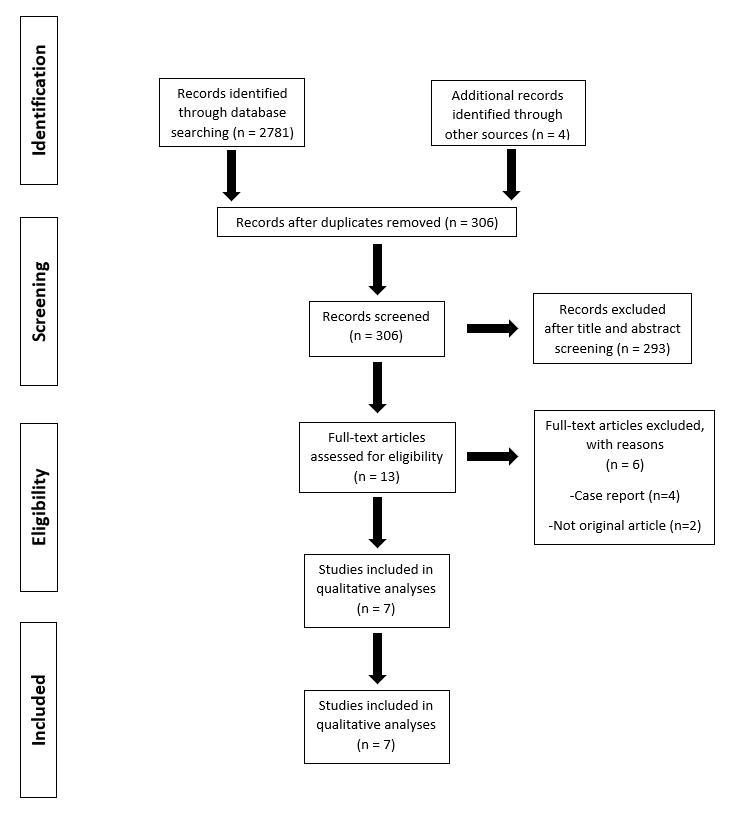  **Supplemental Figure 2.** Prisma Flow Diagram |
| --- |

| **Supplemental Table 3. Baseline Characteristics of Patients** | | | | | |
| --- | --- | --- | --- | --- | --- |
| **RCTs** | **COMPASS** | | | **AFIRE** | |
| **Characteristic** | Rivaroxaban plus Aspirin (N=9152) | Rivaroxaban Alone (N=9117) | Aspirin Alone (N=9126) | Rivaroxaban Monotherapy (N=1107) | Combination Therapy (N=1108) |
| Age — yr | 68.3±7.9 | 68.2±7.9 | 68.2±8.0 | 74.3±8.3 | 74.4±8.2 |
| Female sex — no. (%) | 2059 (22.5) | 1972 (21.6) | 1989 (21.8) | N/A | N/A |
| Male sex — no. (%) | N/A | N/A | N/A | 875 (79.0) | 876 (79.1) |
| Body-mass index† | 28.3±4.8 | 28.3±4.6 | 28.4±4.7 | 24.5±3.7 | 24.5±3.7 |
| Race or ethnic group — no. (%)† |  |  |  |  |  |
| White | 5673 (62.0) | 5672 (62.2) | 5682 (62.3) | N/A | N/A |
| Black | 76 (0.8) | 94 (1.0) | 92 (1.0) | N/A | N/A |
| Asian | 1451 (15.9) | 1421 (15.6) | 1397 (15.3) | N/A | N/A |
| American Indian or Alaska Native | N/A | N/A | N/A | N/A | N/A |
| Other or unknown | 1952 (21.3) | 1930 (21.2) | 1955 (21.4) | N/A | N/A |
| Previous stroke — no. (%) | 351 (3.8) | 346 (3.8) | 335 (3.7) | 148 (13.4) | 175 (15.8) |
| Previous myocardial infarction — no. (%) | 5654 (61.8) | 5653 (62.0) | 5721 (62.7) | 384 (34.7) | 393 (35.5) |
| Diabetes — no. (%) | 3448 (37.7) | 3419 (37.5) | 3474 (38.1) | 461 (41.6) | 466 (42.1) |
| Type of atrial fibrillation — no. (%) |  |  |  |  |  |
| Paroxysmal | N/A | N/A | N/A | 596 (53.8) | 580 (52.3) |
| Persistent | N/A | N/A | N/A | 164 (14.8) | 175 (15.8) |
| Permanent | N/A | N/A | N/A | 347 (31.3) | 353 (31.9) |
| Current smoker — no. (%) | N/A | N/A | N/A | 146 (13.2) | 146 (13.2) |
| AF: Atrial fibrillation, PCI: Percutaneous Coronary Intervention, CABG: Coronary artery bypass graft, CAD: Coronary artery disease, PAD: Peripheral artery disease, GFR: Glomerular filtration rate, DAPT: Dual antiplatelet therapy, MI: Myocardial infarction, ISTH: International Society on Thrombosis and Haemostasis (ISTH), DOAC: Direct oral anticoagulant, VTE: Venous thromboembolic disease, DVT: Deep vein thrombosis, PE: Pulmonary embolism, AFL: Atrial flutter, MACE: Major adverse cardiac events, ACS: Acute coronary syndromes, CRNMB: Clinically relevant non-major bleeding | | | | | |

| **Supplemental Table 3. Baseline Characteristics of Patients (continued)** | | | | | | | | | | | | | | | |  |
| --- | --- | --- | --- | --- | --- | --- | --- | --- | --- | --- | --- | --- | --- | --- | --- | --- |
| **Observational  Studies** | **Schaefer, 2019** | | **Tinkham, 2019** | | | **Davidson, 2014** | | | | **Steinberg, 2013** | | | **Ruiz, 2019** | | |  |
|  |  |  |  |  |  |  |  |  |  |  |  |  |  |  |  |  |
| **Characteristic** | DOAC  (N=639) | DOAC+ASA  (N=639) | Overall  (N=407) | DOAC+APT  (N=78) | DOAC monotherapy (N=329) | No NSAID Use (N=6362) | Any NSAID Use  (N= 1884) | No Aspirin Use  (N = 7044) | Any Aspirin Use (N=1202) | Overall (N=7347) | OAC Alone (N=4804) | OAC+ASA (N=2543) | Whole Population (N=2361) | Without ATP therapy (N=2216) | With  ATP  (N=145) |  |
| Age,  mean (SD),y | 71.6 | 71.8 | 63.7  (16.0) | 63.4  (16.2) | 63.2  (16.0) | 58  (17) | 55  (17) | 56  (17) | 64  (15) | 75  (68–82) | 76  (68–82) | 75  (67–81) | 76 ± 9 | 76 ± 10 | 77 ± 7 |  |
| Female sex  no. (%) |  |  |  |  |  |  |  |  |  | 43% | 47% | 34% | 1260  (53) | 1201  (54) | 59  (41) |  |
| Male sex  no. (%) | 357  (55.9) | 357  (55.9) | 228  (56.0) | 43  (55.1) | 185  (56.2) | 3558  (55.9) | 942  (50.0) | 3811  (54.1) | 689  (57.3) |  |  |  |  |  |  |  |
| BMI, mean (SD), kg/m2 | N/A | N/A | 29.1  (5.3) | 30.7  (5.3) | 28.4  (5.2) | N/A | N/A | N/A | N/A | N/A | N/A | N/A | N/A | N/A | N/A |  |
| Hypertension no.(%) | N/A | N/A | 297  (73) | 59  (75.6) | 238  (72.3) | N/A | N/A | N/A | N/A | 85 | 83 | 87 | 2057  (87) | 1922  (87) | 135  (93) |  |
| Atrial fibrillation no.(% | 475  (74.3) | 476  (74.5) | 190  (46.7) | 38  (48.7) | 152  (46.2) | N/A | N/A | N/A | N/A | N/A | N/A | N/A | 1208  (52) | 1134  (52) | 74  (54) |  |
| Heart Failure no.(%) | N/A | N/A | N/A | N/A | N/A | N/A | N/A | N/A | N/A | 34 | 31 | 39 | 436  (19) | 404  (18) | 32  (22) |  |
| Diabetes  no. (%) | N/A | N/A | 78  (19.2) | 21  (26.9) | 57  (17.3) | N/A | N/A | N/A | N/A | 30 | 28 | 35 | 735  (31) | 659  (30) | 76  (52) |  |
| Current smoker no. (%) | N/A | N/A | N/A | N/A | N/A | N/A | N/A | N/A | N/A | 48 | 45 | 54 | 140  (5.9) | 131  (5.9) | 9  (6.2) |  |
| AF: Atrial fibrillation, PCI: Percutaneous Coronary Intervention, CABG: Coronary artery bypass graft, CAD: Coronary artery disease, PAD: Peripheral artery disease, GFR: Glomerular filtration rate, DAPT: Dual antiplatelet therapy, MI: Myocardial infarction, ISTH: International Society on Thrombosis and Haemostasis (ISTH), DOAC: Direct oral anticoagulant, VTE: Venous thromboembolic disease, DVT: Deep vein thrombosis, PE: Pulmonary embolism, AFL: Atrial flutter, MACE: Major adverse cardiac events, ACS: Acute coronary syndromes, CRNMB: Clinically relevant non-major bleeding | | | | | | | | | | | | | | | | |

|  |
| --- |
